# Organ-on-a-chip technologies for biomedical research and drug development: A focus on the vasculature

**DOI:** 10.1002/SMMD.20220030

**Published:** 2023-02-24

**Authors:** Diosangeles Soto Veliz, Kai-Lan Lin, Cecilia Sahlgren

**Affiliations:** 1 Faculty of Science and Engineering, Cell Biology, Åbo Akademi University, Turku, Finland; 2 InFLAMES Research Flagship Center, Åbo Akademi University, Turku, Finland; 3 Turku Bioscience Center, Åbo Akademi University and University of Turku, Turku, Finland; 4 Department of Biomedical Engineering, Eindhoven University of Technology, Eindhoven, the Netherlands; 5 Institute for Complex Molecular Systems (ICMS), Eindhoven University of Technology, Eindhoven, the Netherlands

**Keywords:** biomedical, microphysiological systems, models, organ-on-a-chip, vasculature

## Abstract

Current biomedical models fail to replicate the complexity of human biology. Consequently, almost 90% of drug candidates fail during clinical trials after decades of research and billions of investments in drug development. Despite their physiological similarities, animal models often misrepresent human responses, and instead, trigger ethical and societal debates regarding their use. The overall aim across regulatory entities worldwide is to replace, reduce, and refine the use of animal experimentation, a concept known as the Three Rs principle. In response, researchers develop experimental alternatives to improve the biological relevance of in vitro models through interdisciplinary approaches. This article highlights the emerging organ-on-a-chip technologies, also known as microphysiological systems, with a focus on models of the vasculature. The cardiovascular system transports all necessary substances, including drugs, throughout the body while in charge of thermal regulation and communication between other organ systems. In addition, we discuss the benefits, limitations, and challenges in the widespread use of new biomedical models. Coupled with patient-derived induced pluripotent stem cells, organ-on-a-chip technologies are the future of drug discovery, development, and personalized medicine.

## 1 Introduction

Current biomedical models lack the biological complexity and responses of the human body, creating a gap between experimental research and clinical outcomes. Failure to translate experimental results is the most pressing challenge in the pharmaceutical industry. Behind every new drug in the market, there are decades of research involved, millions to billions worth of investment, and plenty of failed drugs left at clinical phase trials.^1^ Furthermore, increasing evidence shows that, despite of physiological similarities, animal models are poor predictors of drug toxicity in humans.^2^ In this sense, a drug labeled “safe” in animal testing may prove toxic to humans in clinical trials. Alternatively, a drug falsely labeled as “toxic” could be a useful therapeutic agent. In few cases, drug toxicity, most commonly hepatic or cardiovascular, only appears after long-term use in humans, leading to sudden withdrawals after approval by regulatory entities. Hence, drug development is close to stagnant, and accurately predicting the reaction of the body to different stimuli under physiological and pathological conditions is crucial for the continuous evolution of medicine. Only biomedical models of improved biological relevance will push the field forward.

This review summarizes current biological research models and expands on Organ-on-a-Chip (OOC) technologies. Briefly, we divide recent literature in terms of different human organ systems, and we further focus on the vasculature. We highlight the key features of the vasculature in the human body, and how these features translate to current needs and limitations of OOC models of the vasculature. The intention is to provide the state-of-the-art and a future perspective on the current challenges to what may be the next-generation research models for understanding disease and drug development.

### 1.1 Current in vivo models

Since Ancient Greece, animal models became the standard tool to understand human anatomy and physiology. In many ways, we owe the modern status of biomedical field to animal modeling and experimentation. The past century had a series of milestones, such as the development of genetic tools, which propelled the use of animals, such as mice, in biomedical research.^3,4^ However, ethical concerns regarding animal welfare and validity of results encouraged the scientific community to restrict the use of animal models. Hence, the Three R’s of animal research was introduced by Russel and Burch in 1959.^5^ Replacement, reduction, and refinement of animal experimentation are the foundations of this principle, and the conceptualization continues to expand since it was first published.^6^

**Figure 1:**
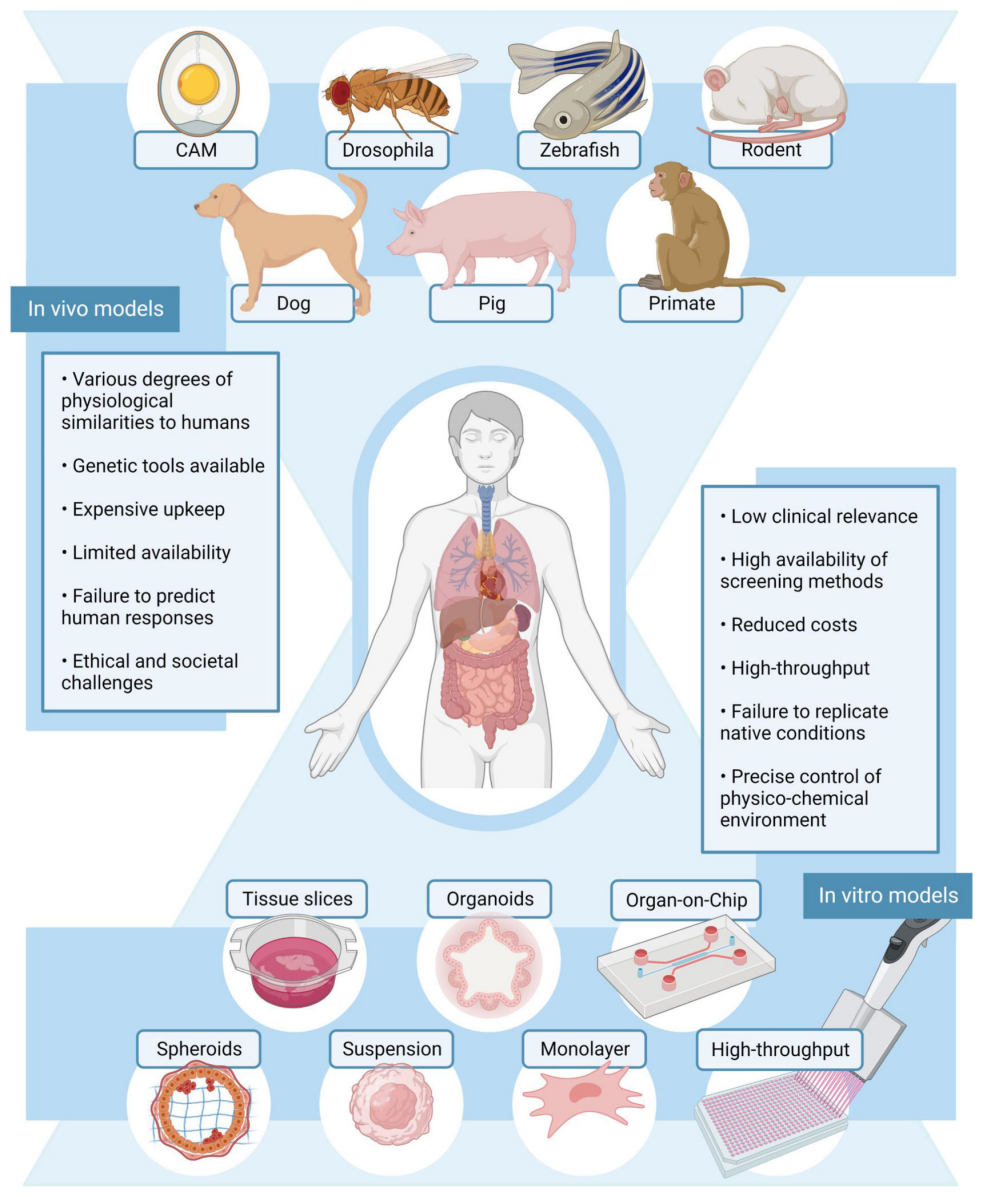
Current in vivo and in vitro models in human research and their highlights. In vivo model examples include the following models: chick chorioallantoic membrane (CAM), *Drosophila melanogaster* (fruit fly), zebrafish (*Danio rerio*), rodents, dogs, pigs, and primates. In vitro models, from low to high biological relevance, include traditional cell culture (suspension or monolayer), high throughput, spheroids, organoids, tissue slices, and organ-on-a-chip technologies. *Source*: Created with BioRender.com.

Figure 1 exemplifies the animal models in modern biomedical research. The choice of model for research purposes depends on the specific biological problem to address and context-dependent criteria, such as access, tractability, resourcing, economies, and promise.^7^ Regardless, often the choice of the model organism is down to”convenience”.The chicken chorioallantoic membrane system is preferred for the in vivo study of blood vessels^8^ and human tumor growth.^9^ The model benefits from the vascularization, multicellular components, and extracellular matrix features from the fertilized chicken egg. In addition, tumor growth is fast, there are less ethical considerations, and the system upkeep is less expensive than other models. *Drosophila melanogaster*, or fruit fly, is 60% homologous to humans in terms of genome, and in the past decade, its use in developmental^10^ and cancer research^11^ has exponentially increased. Short generation time, availability of genetic tools, low maintenance cost, and few ethical considerations are some of the benefits of this organism. Zebrafish (*Danio rerio*) is physiologically and genetically similar to humans since it has a brain, digestive track, musculature, vasculature, and innate immune system. As a biomedical model, it is easy to manipulate genetically, requires low space and maintenance cost, and has a rapid development.^12^ Furthermore, during the embryonic stage, zebrafish is optically clear and can survive up to 3 days without blood flow.^13^ This feature is ideal for the study of mechanosensitive or flow-responsive genes.^14^ Zebrafish embryos are currently key to understanding vascular development,^15^ cardiovascular diseases,^16^ and tumor angiogenesis.^17^ Altogether, these models facilitate the simplified study of human physiology; however, mammalian models are the closest representatives of human complexity.

Today, by far, rodents are the most used model organism, yet not always suitable for translational research as the system fails to replicate several human responses. The mouse model, for example, is a cost-efficient model whose genome is 99% similar to the human genome. Plenty of genetic and molecular engineering tools already exist for this system, and the small size makes it compatible for large-scale studies in medical research.^18,19^ In contrast, large animals recapitulate to a better extent human anatomy. Dogs are a powerful model organism for gene therapies, aging, regenerative medicine, and mortality research as we closely share environmental factors.^20,21^ Swine models provide a better understanding of human disease models and are an optimal model for xenotransplantation and surgical training as their organs are of similar size to human organs.^22,23^ Sheep, as a large-animal model, shares hemodynamic flow parameters with similar anatomy to the human body and is a standard preclinical model for the testing of the efficacy and safety of new drug formulations and vaccines.^24^ Nonhuman primates share biological, clinical, and behavioral (cognitive and societal) features with humans; thus, they are the most vital animal model.^25,26^ However, due to the vast amount of similarities, animal welfare considerations within experimentation are the highest in this model. Overall, mammalian model organisms increase the ethical and societal concerns, upkeep is expensive, and there is limited availability. As such, the use of large mammalians within the scientific community is decreasing, while the focus turns to alternative in vitro models.

### 1.2 Current in vitro models

Since the early 1900s, mammalian cells were cultured on flat plastic surfaces, where cells formed an adherent monolayer. Later, in the 1950s, suspension culture of mammalian cells was developed where cells grow suspended in the cell culture media. Suspension culture was rapidly adopted in the biotechnology industry for large-scale production.^27^ In suspension, cells grow as single cells or small aggregates in culture flasks, rotating-wall vessels, or reactors.^28,29^ Both classical approaches (adherent and suspension) are the most used models within in vitro research due to their simplicity, low cost, and high availability of functional assays and monitoring methods. In addition, classical culture models can be coupled to automated systems for high-throughput drug screening. However, they lack the characteristics of the native extracellular environment, and experimental results often fail to translate to clinical outcomes. Surface coating of extracellular matrix (ECM) components is one way to improve the relevance of adherent culture, yet not sufficient for a valid biomedical model.

3D culture aims to mimic the in vivo environment and behavior of cells.^30^ First attempts at 3D culture were made in 1970s,^31^ but it was only in early 2000s that the approach gains exponential popularity. The model is still in its infancy and requires optimization of the cell culture conditions. However, the results already show that cellular morphology is preserved, and it is possible to replicate cell–cell and cell–ECM interactions. 3D culture is particularly useful in cancer biology,^32^ facilitating the screening of biomarkers and therapies.

In general, in vitro models rely mostly in established cell lines or primary cell lines. Established cell lines are stored in bioresource centers where they are well characterized and used routinely in research. In contrast, primary cell lines are isolated from donors’ material so that they are biologically relevant but are often costly with a short passage span. However, 3D spheroids and organoids models can be produced from pluripotent stem cells. Organoids will be key biomedical models in the future as they are patient-specific. Alternatively, some researchers use tissue slices directly from living organisms to understand human diseases. Tissues are a complex heteroge-neous model and of limited availability. Instead, organ-on-a-chip technologies combined with other in vitro models can recapitulate in vivo environments and facilitate real-time monitoring and assessment in controlled conditions.

## 2 Engineering Microphysiological Systems

OOC technologies are the result of an interdisciplinary approach to life sciences, combining expertise from cell biology, engineering, physical, and chemical sciences. These models, also known as microphysiological systems (MPS), intend to recapitulate the biological functionality of human physiology. OOC models emerged from recent advancements in microfluidics-based devices known as lab-on-a-chip.^33^ The miniaturization of functional assays rapidly found a wide range of applications in diagnostics, therapeutics, biosensors, and then cell culture. Benefits included small working volumes, faster reaction times, low-cost, and increased precision and control over the experimental design. These advantages appealed to the scientific community in life sciences, where control over the cellular environment was needed. Other approaches to MPS, outside microfluidics, include organoids and 3D bioprinting.^34^ Organoids are fabricated through hanging drop or microwell array landing methods. Hence, organoids benefit from self-organization and self-renewal of the cellular environment and lead to similar composition, architecture, and functionality as the organ of origin. In contrast, 3D bioprinting builds custom-made architectures similar to human organs or tissues in a high-throughput manner, using inkjet, extrusion, or laser-assisted bioprinting.

OOCs are mostly manufactured through soft-lithography with polydimethylsiloxane (PDMS), a technology first developed by the Whiteside’s group. Other emerging fabrication methods include injection molding, hotembossing, viscous finger patterning, 3D printing, and sacrificial bioprinting.^35–39^ Table 1 briefly describes advantages and disadvantages of most common manufacture methods of OOC technologies. In terms of materials, PDMS is widely used in microfluidics because of its optical transparency, controllable elasticity, gas permeability, and biocompatibility. However, recent studies show that PDMS affects long-term cell culture through small molecule absorption^40^ or leaching of PDMS oligomers.^41^ Therefore, alternative materials, such as thermoplastic polymers, are on the rise in the OOC field.^42^

OOCs mimic specific organ functions with microfluidic channels, cell culture compartments, membranes, perfusion systems, and the addition of various bio-materials that simulate the extracellular environment. Biomechanical stimuli, equivalent to those inside our bodies, are easily integrated to OOCs and are crucial for biological relevance as mechanobiology determines tissue development and function.^43^ In this section, we highlight literature that reviews the state-of-the-art of OOC technologies for each organ system before taking an in-depth look at the vasculature.

### 2.1 Musculoskeletal system

Musculoskeletal injuries are the major cause for disability worldwide; yet most common ailments, such as arthritis and other pain-related conditions, were, for a long time, extremely difficult to simulate inside the laboratory. The musculoskeletal system supplies mechanical support to the human body and includes muscles, bones, joints, ligaments, and tendons. The main challenge within this system is to mimic the wide range of complexity, highly organized structure, and load bearing capacity of these tissues. Recent literature reviews the current efforts to improve our understanding of the musculoskeletal system and its pathologies using OOC technologies.^44–46^ Particularly, the development focuses on OOC models of tendons,^47,48^ joints,^49,50^ and vascularized bone.^51^

### 2.2 Digestive system

The digestive system breaks down nutrients from what we eat and drink to be absorbed and used in different parts of our body. This is the main source of energy inside the human body. Diseases and disorders of the digestive track can be both temporary or long-lasting (chronic). Other than cancer, the most common diseases are acid reflux, irritable bowel syndrome, Crohn’s disease, celiac disease, and lactose intolerance, often detected through invasive diagnostic tests. However, it is the prevalence of gastrointestinal symptoms that affect most the overall population. Modeling of the digestive system inside the laboratory benefits and also the understanding of drug absorption as gastrointestinal safety become increasingly important in drug discovery and development.^52,53^ The main challenges of biological models of the digestive system are recreating enzymatic digestion and the native microbiome. Recent literature reviews the current efforts to improve our understanding of the digestive system functions and pathologies using OOC technologies,^54,55^ including salivary glands,^56^ stomach,^57,58^ intestine,^59–61^ and liver.^62,63^

### 2.3 Respiratory system

The respiratory system oversees breath, air temperature, and humidity regulation, removing waste gases and protecting the airways. Dysregulation of the respiratory system leads to uncurable chronic respiratory diseases, such as chronic obstructive pulmonary disease (COPD), asthma, occupational lung diseases, and pulmonary hypertension. COPD alone is the third leading cause of death worldwide, and the current focus is on the early diagnosis, treatment, and prevention. Long-term exposure to harmful gases and particles is the major cause of COPD, and therefore, OOC technologies in the respiratory system often anchor in understanding and assessing the toxicology of airborne particulates.^64^ Nowadays, literature of OOC technologies for the respiratory system^65,66^ includes the nasal cavity,^67^ bronchial airways,^68^ and lungs.^69–71^

### 2.4 Urinary system

The urinary system filters blood and drains all wastes through urine, a by-product consisting of waste and water. Most common urologic diseases or disorders include kidney stones, urinary tract infection, enlarged prostate, and incontinence. OOC technology efforts for this organ system are limited and mainly focus on drug efficacy evaluation and urology cancer research,^72^ but also include specifically kidneys^73,74^ and bladder.^75,76^

### 2.5 Reproductive organs

The reproductive system takes care of producing offspring mainly through the transport and sustenance of egg and sperm cells. Replicating the physiology and pathology of the reproductive system inside the laboratory is a key aspect of human health as it involves fertility and sexually transmitted infections. OOC technologies attempt to replicate the female reproductive system, which are summarized by Stejskalová et al.^77^ and Young and Huh in 2021,^78^ and are expanded recently to the vagina,^79^cervix,^80^ and pregnancy-related models.^81^ In contrast, the male reproductive system is less represented in OOC technologies^82^ with very few models of prostate gland,^83^ epididymis,^84,85^ spermatogenesis,^86^ and testis.^87,88^

### 2.6 Endocrine system

The endocrine system is formed by a tightly regulated network of organs and glands tasked with the production and release of hormones. In turn, hormones regulate the body’s metabolism, reproduction, growth, development, emotions, sleep, and the response to injury and stress. In research, both in silico^89^ and OOC models^90^ are ideally integrated to predict complex hormone dynamics. However, OOC technologies of the endocrine system are poorly represented^59^ and limited to the parathyroid glands,^91^ pancreas,^92,93^ and neuroendocrine research.^94^

### 2.7 Nervous system

The nervous system is in charge of communication within the body, acting as a command center between the brain and the body. It consists mainly of the central nervous system and the peripheral nervous system and may include the sensory organs. Out of all the components, the brain is the main player of the nervous system, and the major obstacle in research translation as it contains the blood-brain barrier (BBB). OOC models are able to replicate key aspects of the BBB microenvironment, such as mechanical queues and cell–cell interactions.^95^ In the past decade, OOC models highlight the importance of a hierarchical multidimensional structure and the use of organoids for the recapitulation of the native nervous system.^96^ Other specific models of the nervous system attempt to mimic the bone marrow,^97^ nerves,^98,99^ and most recently, sensory organs.^100,101^

### 2.8 Integumentary system

The integumentary system forms the outer layer of the human body and constitutes the first line of defense against the environment, including injuries and pathogens. It is also a regulator of body temperature and bodily fluids. This organ system is composed of the skin, nails, hair, and exocrine glands in the human body. However, the contrasting differences and diversity in the composition of some of these organs^102,103^ limit the design of a universal OOC for the integumentary system. The main OOC models for specific organs in this system aim to replicate the skin^104,105^; while mammary glands, for example, are often only studied in the context of branching morphogenesis^106^ or breast cancer.^107^ Replicating skin is of high relevance for skin-targeted delivery systems (topical, dermal, and transdermal approaches).^108^ Hence, most skin models focus on the efficient study of skin–drug interactions and toxicology, where the transport properties and the intrinsic heterogeneity of the skin are key for the validation of drugs and cosmetic compounds.^109–111^

### 2.9 Circulatory system

The circulatory system consists of a complex network of vessels. In the circulatory system, the blood vessels transport blood to and from the heart; while in the lymphatic system, lymph vessels regulate fluid homeostasis in tissues. The lymphatic system is closely related to the immune system, and OOC models of this vessel network are fundamental to both pharmacological and toxicological applications^112^ and to cancer research.^113^ In contrast, OOC models of the cardiovascular system highlight research in cardiovascular pathologies^114^ and drug discovery and development.^115^ The heart is the center of the cardiovascular system, followed by the components of the systemic circulation (arteries, capillaries, and veins). The following sections focus on the main features of the systemic circulation needed in OOC models, the state-of-the-art within research, and current challenges.

## 3 Mimicking The Vasculature

**Figure 2:**
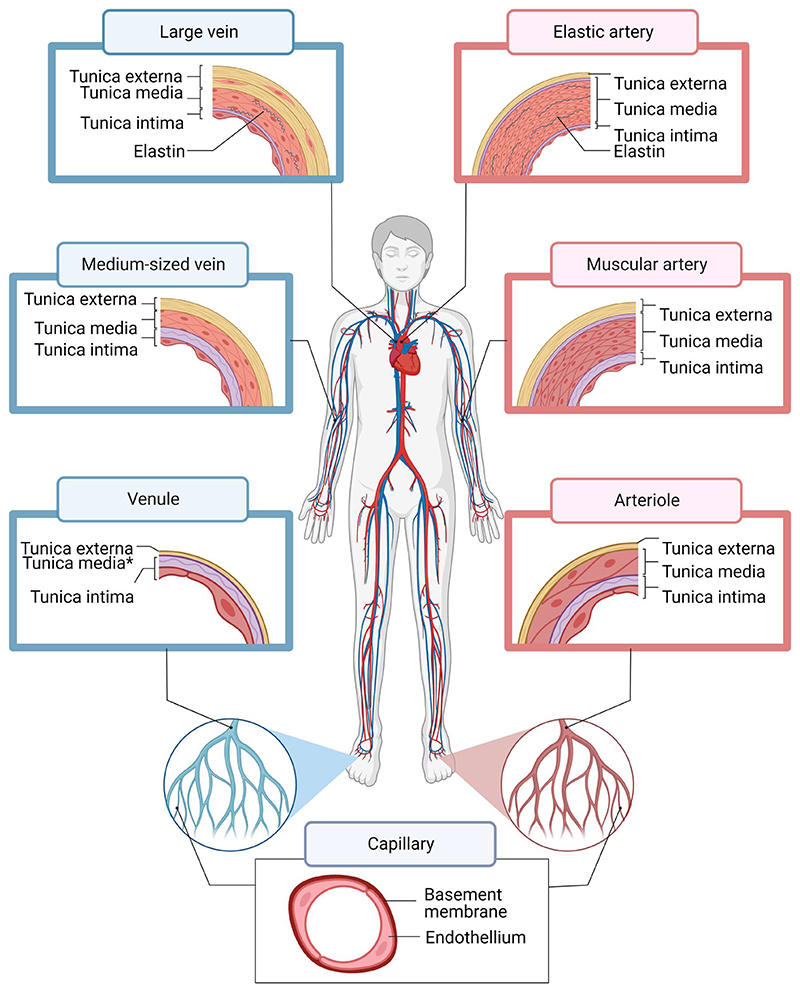
Schematic representation of the arterial and venous networks. Blood circulation starts in the heart where blood is pumped with a highly pulsatile flow. The arterial system stabilizes the blood flow and distributes it throughout the body until the capillary bed, where diffusion takes over transport of nutrients and waste. Lastly, the venous system transports back the blood to the heart. The cross-sections showcase the structure of each blood vessel wall, including the inner layer (tunica intima), the middle layer (tunica media), and the outer layer (tunica externa or adventitia). In the case of venules, a clear tunica media may not always be present. *Source*: Adapted from “Arterial Blood Vessels”, by BioRender.com (2023). Retrieved from https://app.biorender.com/biorender-templates

The vasculature has mainly four functions inside the human body^116^: (a) regulate and transport nutrients and waste, (b) upkeep the immune system, (c) maintain environmental variables, such as temperature and pH, and (d) control homeostasis. These functions start from the pumping of the heart and continue with the circulation of blood throughout the body with the arterial and vein networks. Figure 2 summarizes the anatomy of the circulatory network of the human body. Briefly, arteries convert the highly pulsatile blood flow from the heart into stable flow all the way until the capillary bed, where diffusion becomes the main mode of transport; then, the venous system pumps the blood back to the heart. This process is known as the systemic circuit, and it is followed by the pulmonary circulation^117^ in which the oxygen poor-blood is reoxygenated through a loop between the heart and the lungs (using pulmonary vasculature) before reinsertion to the systemic circulation. The continuous regulation of the blood flow throughout the body makes the arterial system a pressure reservoir that requires various degrees of wall elasticity.^118^ Arteries closer to the heart are “elastic,” while those branching further away become “muscular”. The ratio between the arterial wall components determines the elastic or muscular behavior as seen in the cross-section representation of each blood vessel type. The structure of the blood vessel wall consists of the tunica intima, the tunica media, and the tunica externa.^119^ The innermost layer is the tunica intima and provides a smooth lining of endothelial cells and elastic tissues for the blood to flow. The middle layer is the tunica media, which consists of smooth muscle cells organized in concentric rings and elastic fibers ready to expand and contract according to the blood flow. Lastly, the tunica externa (or adventitia) is made of connective tissues, such as collagen and elastin, and its main role is to anchor the blood vessels to the surrounding tissue while preventing overexpansion due to blood pressure.

The intrinsic yet complex composition of the different blood vessels is one of the greatest challenges to overcome when mimicking the vasculature. The solution, often, is to simplify the multicomponent vasculature to models with a single cell type and a homogenous extracellular matrix that hardly represent the native environment. Table 2 shows average parameters for each blood vessel type, including diameter, wall thickness, and hemodynamic cues (shear rate and shear stress).^120–123^ The broadness of each parameter highlights once more the differences between blood vessels and the need to consider their anatomical characteristics in research models.

### 3.1 Flow-induced mechanical stimuli in the vasculature

In terms of anatomical structure of the blood vessels, only the tunica intima is in contact with blood. However, the mechanical stimuli derived from the blood flow translate across the whole blood vessel wall. According to Table 2, blood flows initially through arteries of approximately 25 mm in diameter, all the way down to vessels of less than a millimeter in diameter. The reduction in diameter is significant and occurs in a relatively short distance inside the body, actively influencing the mechanical forces exerted by blood circulation on the vessel wall. Blood flow and biofluid mechanics in the arterial and venous system are extensively discussed elsewhere,^124^ including the assumption of steady flow for blood flow. This assumption is currently a standard within research, as it simplifies the vascular model; however, in reality, blood flow does not have a constant flow rate as it shifts between pulsatile flow and turbulence in time depending on the location and/or pathology. Often, the fluctuation in blood flow is discussed as oscillatory blood flow, where time variations of flow conditions in specific spatial gradients in the vessel wall are of interest.

**Figure 3:**
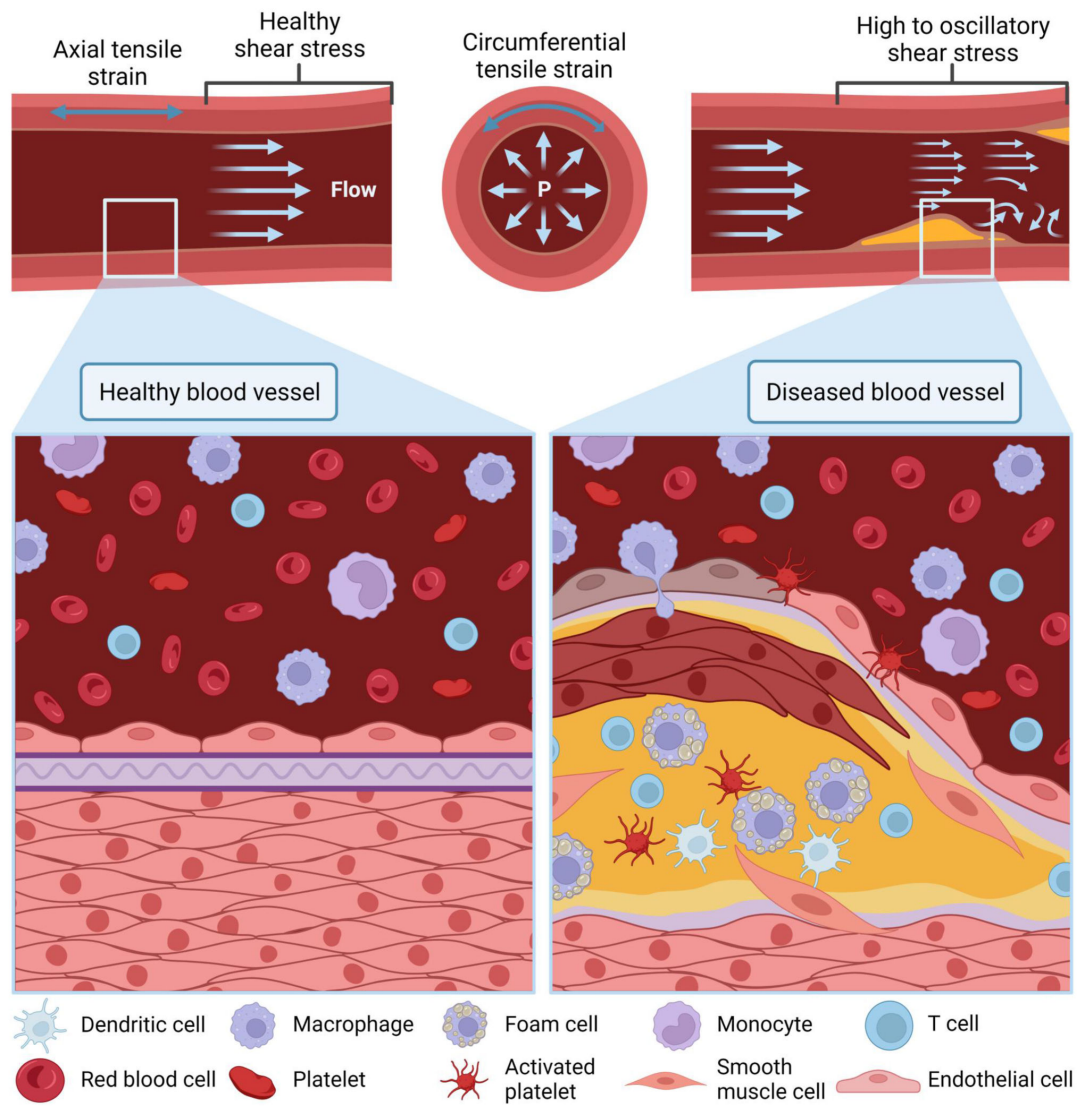
Schematic representation of physiological (healthy) and pathological (diseased) conditions of a blood vessel. The figure illustrates also the velocity profile of blood flow across both conditions that lead to shear stress, and the circumferential blood pressure experienced as tensile strain on the blood vessel. In the healthy blood vessel, the endothelium lining is intact. In contrast, in a diseased blood vessel, there is an endothelial injury represented by darkened endothelial cells, where components leak through and build up to plaque formation. The plaque is a multicellular deposit that upon rupture leads to thrombosis and tissue infarctions. *Source*: Created with BioRender.com.

Figure 3 illustrates the environment and blood flow during physiological and pathological conditions. At all times, during blood flow, blood vessels experience many types of fluid forces, including shear stress and tensile strain. When flowing, blood exerts a tangential force over the endothelium lining of the vessel wall, and the resulting mechanical force is known as shear stress. Under shear stress, endothelial cells may undergo morphological changes, remodel the extracellular matrix, or trigger pathological conditions.^125–127^ At the same time, the rest of the blood vessel undergoes tensile strain due to the blood pressure. Tensile strain occurs when fluid flow exerts forces on a surface to stretch the material. Inside a blood vessel, smooth muscle cells are the most sensitives to circumferential tensile strain. Tensile strain, also known as cyclic strain, triggers morphological reorganization,^128^ phenotypic changes through Notch signaling,^129^ and alignment^130^ of vascular smooth muscle cells. Overall, mechanical stimuli, such as shear stress and strain, maintain and tightly regulate homeostasis^131^ and the endothelial barrier^132^ through mechanotransduction. Abnormal mechanical cues are the molecular basis of cardiovascular diseases.^133^

In a healthy vessel, the endothelium lining is intact, blood flows uninterruptedly, and all blood components are transported throughout the body. In pathological conditions, there is often endothelium barrier rupture and accumulation or malformations within the blood vessel that obstruct the passage of blood. The obstruction in time leads to high or oscillatory shear stress areas that dysregulate the blood vessel wall and lead to deadly cardiovascular diseases, such as coronary artery disease.^119^ The best representation of this scenario is atherosclerosis. Atherosclerosis is an inflammatory disease that affects the inner lining of arteries, reducing their ability to stretch.^134^ It starts by an inflammatory response to an injury to the vessel wall, which leads to wall scarring and a weak endothelium lining. Then, fatty components in blood accumulate through the broken endothelial barrier, followed by immune components and calcium ions. Slowly, the accumulation leads to a plaque formation that narrows the arteries reducing arterial perfusion. Over time, vascular smooth muscle cells are also recruited to the plaque core and become a major cell type in the atherosclerotic plaque.^135^ Eventually, due to mechanical stress, the plaque becomes unstable and erodes or ruptures resulting in thrombosis and acute tissue infarctions. Therefore, mimicking both the vessel wall components and the mechanical forces in the cardiovasculature is key to understanding, preventing, and treating blood vessel dysfunction.

### 3.2 Organ-on-a-chip models of the vasculature

**Figure 4:**
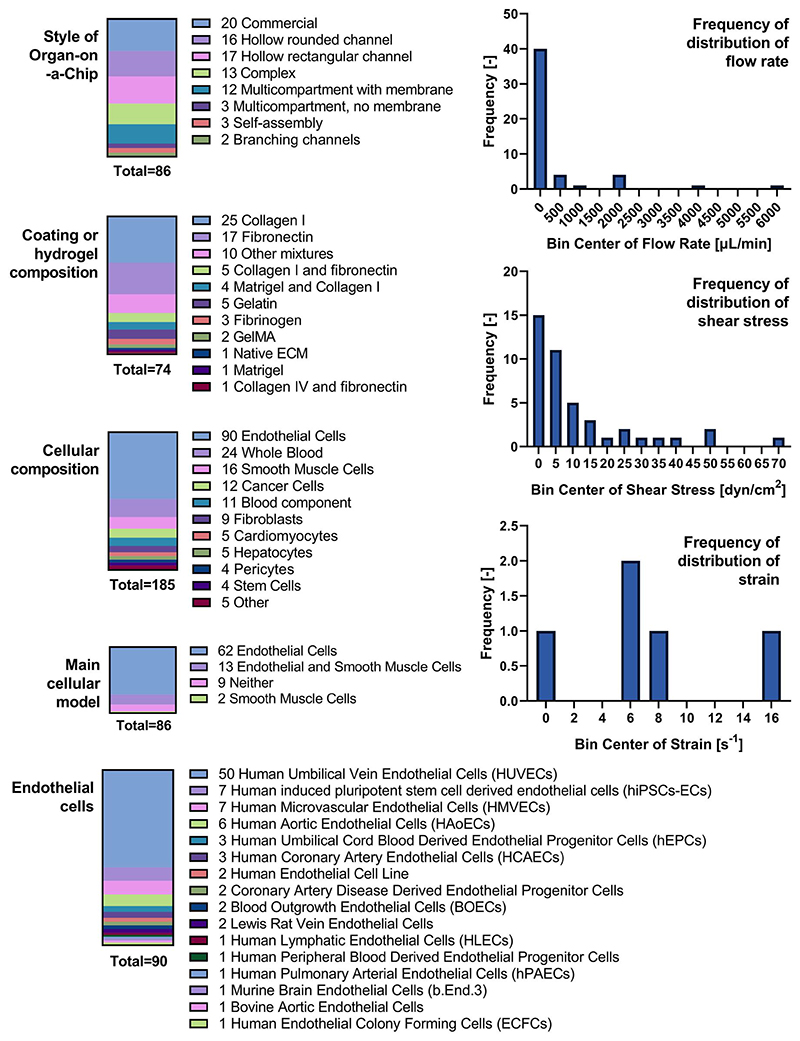
Summary of 86 studies of Organ-on-a-Chip models of blood vessels. Studies were analyzed according to the device design (style), coating or hydrogel composition, cellular composition, main cellular model, and type of endothelial cells used during the study. In addition, experimental parameters of flow were collected, including the flow rate, shear stress, and strain. These parameters are illustrated as frequency bar graphs. Not all 86 studies used flow, nor included all parameters; therefore, the total amount of studies varies per parameter.

OOC approaches to engineer the vasculature have been recently reviewed in the literature,^37,136^ including progress on atherosclerosis models.^137^ Therefore, this review will instead discuss which features of the vasculature are present in the current research and what are the shortcomings as of now. For this purpose, 86 scientific papers were collected (see Table S1) and summarized in parameters, such as (a) style of Organ-on-a-Chip, (b) coating or hydrogel composition, (c) cellular components, (d) flow rate, (e) shear stress, (f) strain, and (g) specific physiological or pathological model. Figure 4 shows briefly the results for some of these parameters. In terms of cellular components, 50 studies (58%) used Human Umbilical Vein Endothelial Cells (HUVECs) as the representative cell line for the endothelial lining. HUVECs are widely used in cardiovascular research due to their ease of use and accessibility; yet recent research shows that endothelial cells are intrinsically heterogeneous and biologically adapt according to the local needs.^138^ Umbilical endothelial cells are constantly exposed to both fetal and maternal hormones and may not reproduce the behavior of endothelial cells in adult arteries or veins. In this sense, human-induced pluripotent stem cell-derived endothelial cells represent an emerging opportunity for MPS.^139^ Other primary endothelial cells often used are human microvascular endothelial cells and human aortic endothelial cells. In terms of immortalized endothelial cell lines, EA. hy926 and ECV304 are the most frequently used.^140^ Ultimately, only 13 models (15%) contained both smooth muscle cells and endothelial cells; while it is a better model than endothelial cells alone, other players, such as the immune component, are currently missing and are needed to increase the biological relevance of the vascular model.

**Figure 5:**
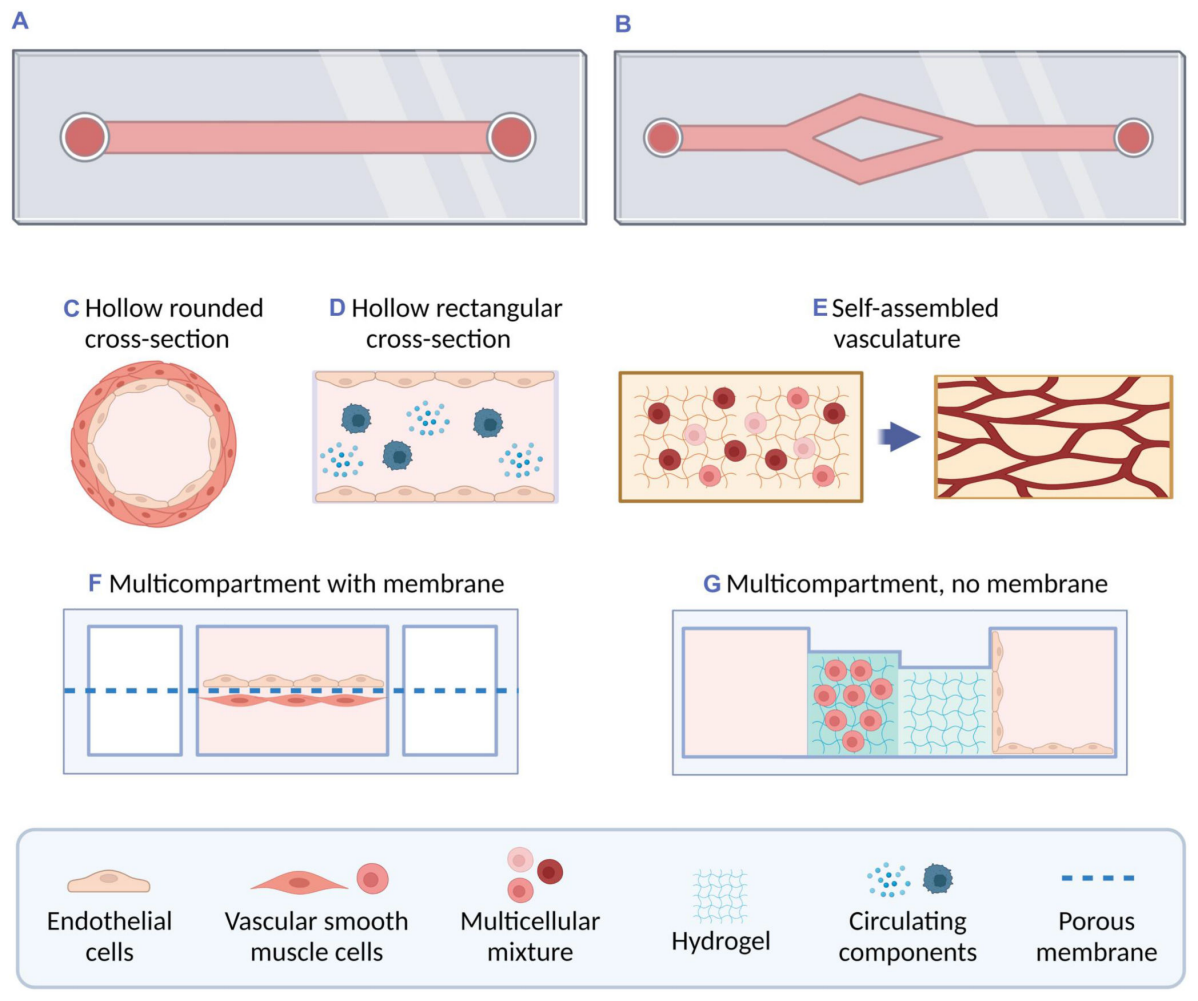
Schematic representation of the most common styles of Organ-on-a-Chip (OOC) technologies for the vasculature. The figure illustrates the general classification of channels: (A) straight single channels and (B) branching channels that often divide into channels of reduced size for a hierarchical structure. Then, the OOC styles are identified according to the cross-section. (C) In hollow rounded channels, endothelial cells and smooth muscle cells can grow layered in the tubular area that resembles the vessel shape. (D) In hollow rectangular channels, endothelial cells usually attach to the bottom and/or top of the channel. In both hollow channels, it is possible to add circulating components in the flow, such as cancer cells or other cellular interactors. (E) Alternatively, single or multicellular components can be mixed with a hydrogel and loaded into a channel to establish a self-assembled vasculature within days of cell culture. (F) and (G) Lastly, OOC models of the vasculature can be designed as multicompartmental devices with or without the membrane to regulate interactions between different cell types and environments. *Source*: Created with BioRender.com.

As for the style of OOC model, in the 86 scientific papers collected (Table S1), commercial or well-established platforms were the most used, such as OrganoPlate,^141^ Ibidi,^142^ AIM Biotech,^143^ AngioChip,^144,145^ Vena8 Endothelial+,^146^ and InVADE.^147^ The rest of approaches were dominated by hollow channels manufactured either by soft-lithography, sacrificial bioprinting, or PDMS casting on polymethyl methacrylate molds. Multicellular models are difficult to integrate in hollow channels unless it occurs through self-assembly. Instead, a multicompartmental structure of the OOC device encourages the use of several cell types. Some of the studies (14%) used the multicompartmental design with a porous membrane to closely monitor and assess the interaction between different cell lines, such as the endothelial and smooth muscle cells. Figure 5 shows a schematic representation of the main styles of Organ-on-a-Chip mimicking the vasculature.^148–154^

Collagen I and fibronectin were the most commonly used components for the extracellular environment. However, on their own, these components do not mimic the complexity of the native extracellular environment. The basement membrane of the vascular wall, for example, has at least 20 proteins with tissue-specific functions, including mainly laminin, type IV collagen, nidogen, perlecan, type XV and type XVIII collagens, fibronectin, heparin sulfate proteoglycan perlecan, and other macromolecules.^155^ In the middle layers of the vascular wall, smooth muscle cells produce primarily elastin and collagen; while in the adventitia, fibroblasts produce an extracellular environment rich in collagen, osteopontin, and fibronectin. As such, the extracellular matrix in blood vessels is a complex network produced by different cell types depending on the region and function and constitutes over half of the vessel wall mass. Some research studies tackle this obstacle by culturing the cells for up to 2 weeks inside the OOC model prior to flow experiments. This way, the cells have enough time to remodel the extracellular matrix. Studies show that the introduction of multicellular cell-laden hydrogels into OOC models, including endothelial cells, results in vasculogenesis and self-assembly into a fully interconnected perfusable microvasculature within a week of cell culture.^156,157^ Furthermore, the recreated vasculature can be implanted in vivo and can lead to extensive host vessel integration with improved blood perfusion.^158^ Lastly, the 3D self-assembly approach benefits pathological OOC models where the dysfunctional remodeling of the extracellular matrix plays a key role on the progression of the disease.

Not every study mimicking the vasculature uses flow, and not every study including flow experiments describes with clarity parameters used, such as flow rate, shear stress, or strain. Therefore, the amount of information available is significantly reduced. Standardization of data and publications is one challenge researchers need to be aware of. From the data available, and in comparison with Table 2, experimental shear stress is most frequently in the range of muscular and elastic arteries despite the lack of biologically relevant dimensions, that is, the wall thickness and diameters of the vessels in the OOC models are significantly smaller than those of the muscular and elastic arteries. As for the cyclic strain, all experimental values are within the change expected for large arteries as well (up to 18%).^159^ However, very few studies (only 6, about 7%) considered cyclic strain in their models and only three studies (3%) in the past decade used both shear stress and cyclic strain during the experiments.^153,160,161^ This is a shortcoming that researchers must address in the vasculature models to come.

## 4 Concluding Remarks and Future Perspective

As research models, OOC technologies have numerous advantages as they have the potential to replicate human physiology. These models can be combined with organoids or patient-derived tissue samples for personalized models and integrated with the latest imaging technologies to extract robust and quantitative information.^162^ The intention with this emerging technology is to recreate specific organ functions inside the laboratory and ultimately combine them into a single body-on-a-chip. Such a device could substitute animal testing and become the next-generation platform for in vitro testing in the pharmaceutical industry,^163–165^ and in space.^166^ As a result, OOC models will deepen pathophysiology understanding,^167^ decrease drug development cost, and improve cancer therapies.^168^

Multiorgan interactions are the next step in OOC technologies; however, this requires further understanding and better modeling of the vasculature that interconnects and transports nutrients and waste between the organs. Physiologically relevant connections between individual organ systems are challenging as models of the vasculature are still in their infancy and we lack a tight regulation of the flow-induced mechanical stimuli, and a universal medium to sustain multiorgan-on-chip technologies.^169^ Furthermore, every organ system has its own set of challenges finding a balance between biological relevance and experimental simplicity.

In this review, we summarized the state-of-the-art of OOC models of the vasculature and assessed that the main shortcomings in current models are the multicellular composition and the representation of both shear stress and cyclic strain simultaneously for a physiologically relevant environment. Vasculature OOC models, on their own, are promising tools as they may provide essential means to study local and distant disease development and treatment, especially cancer initiation and metastasis,^170^ the circulatory system as a biological barrier in drug research, and the evaluation of nanomedicines.^171,172^ Yet, these models remain narrowly used. The main obstacles lie in the accurate representation of physiological and pathological environments.^173,174^ No current OOC model recapitulates the complexity of the human vasculature if physical, chemical, and physiological aspects of healthy and diseased vessels are considered. All of these aspects have a direct effect on drug behavior and are key to a tightly regulated field, such as the pharmaceutical industry. At the same time, increased complexity in the OOC design to simulate the structural dynamics of the native environment can negatively affect reproducibility and large-scale production, while decreasing also ease of handling. The need for multidisciplinary collaboration for a more efficient use of vascular OOC in drug research is another barrier to overcome; however, this is true for most OOC models.^175^

Moreover, there are plenty of challenges to overcome for the widespread adoption of OOC technologies inside the laboratory and within the industry^176^: (a) there is a strong lack of communication between OOC developers and potential users both in academia and industry, (b) there is no widely accepted validation or regulatory framework, (c) there are very few successful business models for the commercialization of OOC technologies, (d) standardization of OCC models is still missing, and at last, (e) high-throughput is limited. Researchers are increasingly aware of these obstacles and roadmaps are currently under investigation, as large governmental units at the European Commission level and the World Health Organization are pushing toward alternatives to animal models.^177,178^

## Supplementary Material

Supporting Information

## Figures and Tables

**Table 1:** Advantages and disadvantages of commonly used manufacture methods of organ-on-a-chip technologies.^35–38^

Method	Advantages	Disadvantages
Soft-lithography/replica molding	High micro-size precision, cost-effectiveness, and versatility to create complex networks	Requires a prefabricated mold, which limits design modifications
Photolithography with polydimethylsiloxane-based soft-lithography	High precision down to nanometers and accurate fabrication of complex structures	Time-consuming, relatively expensive, challenging to recreate rounded cross-sections or tubular geometries
Injection molding	Low cost, ready-made, scalable for mass production, and compatible with high-throughput models	Not suitable for complex designs and functional features, requires a prefabricated mold, and tight regulation of temperature, pressure, and injection rate
Hot embossing	Low cost, ideal for polymeric microstructures with high aspect ratio and micro-pin lamellae, and suitable for most thermoplastic materials	Requires precise regulation of temperature and other parameters for a high-quality surface
Viscous finger patterning	Suitable for hollowed constructs, low cost, and easy fabrication	Not suitable for complex designs and functional features and results in designs with low resolution and low accuracy
3D printing	Low cost, good compatibility to several biomaterials, and provides precise control over designs	Resolution, structure stability, and time consumption vary largely within different techniques and result in inadequate optical transparency
Sacrificial bioprinting	Ideal for lumenized vascular networks, compatible to photocurable hydrogels, high structural integrity with superior mechanical properties, and suitable for hollowed constructs	Long fabrication process, low resolution, and low accuracy

**Table 2:** Average vessel diameter, wall thickness, shear rate, and shear stress for each blood vessel type.^120–123^

Blood vessel	Diameter (mm)	Wall thickness (mm)	Shear rate (s^−1^)	Shear stress (dyn/cm^2^)
Elastic artery (aorta)	25	2	150–250	5–10
Muscular artery	4	1	300	10
Arteriole	0.030	0.006–0.020	1600	54
Capillary	0.008	0.0005–0.001	1300	44
Venule	0.02	0.001–0.002	400	14
Medium-sized vein	5	0.5	100	4
Large vein (vena cava)	30	1.5	>10	>0.5

## Key points

- The current state of organ-on-a-chip (OOC) technologies is highlighted for all organ systems inside the human body with a focus in the vasculature.
- The most important features to be mimicked from the vasculature are recapitulated, such as multicellular components, extracellular matrix, and mechanical stimuli.
- Recent vascular OOC models are summarized in parameters, such as style of OOC, coating or hydrogel composition, cellular components, flow rate, mechanical stimuli, and specific physiological/pathological model.
- This review reports current needs and challenges in vascular OOC models.
